# Successful knowledge translation intervention in long-term care: final results from the vitamin D and osteoporosis study (ViD*OS*) pilot cluster randomized controlled trial

**DOI:** 10.1186/s13063-015-0720-3

**Published:** 2015-05-12

**Authors:** Courtney C Kennedy, George Ioannidis, Lehana Thabane, Jonathan D Adachi, Sharon Marr, Lora M Giangregorio, Suzanne N Morin, Richard G Crilly, Robert G Josse, Lynne Lohfeld, Laura E Pickard, Mary-Lou van der Horst, Glenda Campbell, Jackie Stroud, Lisa Dolovich, Anna M Sawka, Ravi Jain, Lynn Nash, Alexandra Papaioannou

**Affiliations:** McMaster University, 1280 Main Street West, Hamilton, ON L8S 4K1 Canada; University of Waterloo, 200 University Avenue West, Waterloo, ON N2L 3G1 Canada; McGill University, 845Sherbrooke Street West, Montreal, QC H3A 0G4 Canada; Western University, Parkwood Hospital, 801 Commissioners Road East, London, ON N6C 5 J1 Canada; University of Toronto, St. Michael’s Hospital, 30 Bond Street, Toronto, ON M5B 1 W8 Canada; Medical Pharmacies Group Limited, 590 Granite Crt, Pickering, ON L1W 3X6 Canada; Osteoporosis Canada, Suite 301, 1090 Don Mills Road, Toronto, ON M3C 3R6 Canada; Division of Geriatrics, Department of Medicine, McMaster University, Geriatric Education and Research in Aging Sciences (GERAS) Centre, St. Peter’s Hospital, Room 151, 88 Maplewood Avenue, Hamilton, ON L8M 1 W9 Canada

**Keywords:** Fracture, long-term care, Vitamin D, prescribing, knowledge translation

## Abstract

**Background:**

Few studies have systematically examined whether knowledge translation (KT) strategies can be successfully implemented within the long-term care (LTC) setting. In this study, we examined the effectiveness of a multifaceted, interdisciplinary KT intervention for improving the prescribing of vitamin D, calcium and osteoporosis medications over 12-months.

**Methods:**

We conducted a pilot, cluster randomized controlled trial in 40 LTC homes (21 control; 19 intervention) in Ontario, Canada. LTC homes were eligible if they had more than one prescribing physician and received services from a large pharmacy provider. Participants were interdisciplinary care teams (physicians, nurses, consultant pharmacists, and other staff) who met quarterly. Intervention homes participated in three educational meetings over 12 months, including a standardized presentation led by expert opinion leaders, action planning for quality improvement, and audit and feedback review. Control homes did not receive any additional intervention. Resident-level prescribing and clinical outcomes were collected from the pharmacy database; data collectors and analysts were blinded. In addition to feasibility measures, study outcomes were the proportion of residents taking vitamin D (≥800 IU/daily; primary), calcium ≥500 mg/day and osteoporosis medications (high-risk residents) over 12 months. Data were analyzed using the generalized estimating equations technique accounting for clustering within the LTC homes.

**Results:**

At baseline, 5,478 residents, mean age 84.4 (standard deviation (SD) 10.9), 71% female, resided in 40 LTC homes, mean size = 137 beds (SD 76.7). In the intention-to-treat analysis (21 control; 19 intervention clusters), the intervention resulted in a significantly greater increase in prescribing from baseline to 12 months between intervention versus control arms for vitamin D (odds ratio (OR) 1.82, 95% confidence interval (CI): 1.12, 2.96) and calcium (OR 1.33, 95% CI: 1.01, 1.74), but not for osteoporosis medications (OR 1.17, 95% CI: 0.91, 1.51). In secondary analyses, excluding seven nonparticipating intervention homes, ORs were 3.06 (95% CI: 2.18, 4.29), 1.57 (95% CI: 1.12, 2.21), 1.20 (95% CI: 0.90, 1.60) for vitamin D, calcium and osteoporosis medications, respectively.

**Conclusions:**

Our KT intervention significantly improved the prescribing of vitamin D and calcium and is a model that could potentially be applied to other areas requiring quality improvement.

**Trial Registration:**

ClinicalTrials.gov: NCT01398527. Registered: 19 July 2011.

## Background

Effective knowledge translation (KT) interventions are essential to encourage the uptake of evidence-based practices. Ideally, the selection of interventions is guided by evidence of effectiveness and efficiency [1]; however, good evidence is not always available or may not be generalizable from one setting to another. Compared with community or acute care settings, there has been little KT enquiry in long-term care (LTC) homes [2].

LTC homes provide 24-hour nursing care and supervision to residents who often have multiple comorbidities, polypharmacy, and shortened life expectancies. Physicians are often located offsite and engage in collaborative decision making with the care team [3]. Given these characteristics, KT strategies need to be targeted at the interdisciplinary team and be tailored to this unique practice environment [4]. Furthermore, rigorous evaluation is required to ascertain whether KT strategies proven to be effective in other care settings are also effective in LTC.

The Vitamin D and Osteoporosis Study (ViDOS) was a pilot, cluster randomized controlled trial that examined both feasibility and prescribing changes as primary outcomes. Pilot trials are designed to assess a number of feasibility objectives prior to conducting a larger trial [5]. Particularly in situations where there is little previous data to inform the process, pilot trials are considered essential prerequisites that will enhance success of a future, wide-scale trial (*ibid*). Given the considerable resources required to implement such a trial, in addition to examining feasibility outcomes, we appropriately powered the study to assess the statistical significance of our primary prescribing outcome.

The knowledge being translated in the ViDOS intervention was evidence-based osteoporosis and fracture prevention strategies, particularly wide-scale, appropriate vitamin D prescribing. An estimated 60 to 80% of LTC residents have osteoporosis [6], and in Canada, it is estimated that the fracture rate for LTC residents is approximately 2 to 4 times that of similarly aged community-dwelling residents [7]. Meta-analyses demonstrate that vitamin D reduces falls [8], and calcium and vitamin D reduce fractures in LTC residents [9]. Despite strong evidence and acceptance by physicians [10], these strategies are under-utilized in LTC [11,12]. Barriers to implementing appropriate fracture prevention include knowledge gaps [13,14], lack of access to bone densitometry, difficulty in applying risk assessment tools within LTC [14], and a lack of organizational processes and policies that support bone health [15]. Our overall aim was to examine a knowledge translation model for delivering evidence-based practices within LTC homes that utilized a standard method of delivery and also addressed the unique learning needs of each home. Targeting interdisciplinary care teams (including medicine, nursing, rehabilitation, and nutrition), our multifaceted model utilized common professional interventions (for example, educational meetings, audit and feedback) and included elements of quality improvement. Reflecting the critical need to target whole system change when implementing evidence into practice [16], particularly in LTC [17], our intervention emphasized home-level process and policy changes.

The focus of the current article is on prescribing changes; feasibility and fidelity results of the ViDOS trial are published elsewhere [18]. Our primary prescribing objective was to determine if the intervention increased the proportion of all residents prescribed vitamin D ≥800 IU over 12 months. Secondary objectives were to examine the influence of the intervention on 1) calcium prescribing and 2) osteoporosis medication prescribing for high-risk residents.

## Methods

### Trial design and setting

The protocol for this trial (NCT01398527) has been published [19]. The Vitamin D and Osteoporosis Study (ViDOS) was a pilot, cluster randomized control trial (RCT) conducted in 40 LTC homes across the province of Ontario, Canada. In Ontario, there are approximately 630 licensed LTC homes that provide residents with onsite nursing care, 24-hour supervision, or personal support [20].

**Figure 1:**
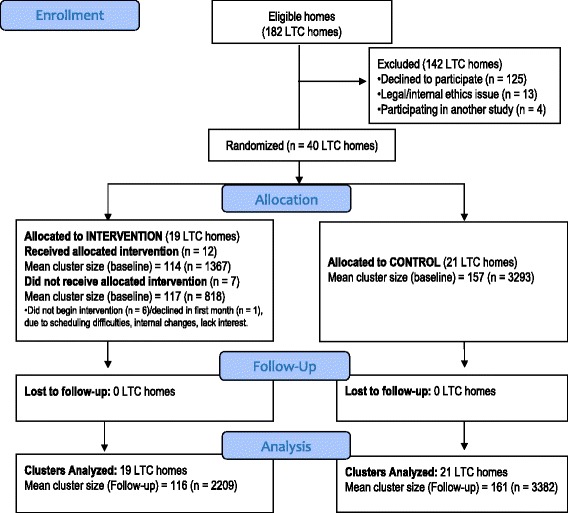
Flow-chart of randomization, allocation, follow-up and analysis.

The trial had two arms: 19 LTC homes were assigned to the intervention group and 21 LTC homes were assigned to a control group (Figure 1). Cluster randomization was chosen due to the natural care clusters (the same professionals caring for residents) in LTC homes. In addition, we wished to minimize the potential for contamination between trial arms given that professionals in the same home may alter their practice for all residents. Furthermore, the ViDOS intervention targeted both individual prescribing physicians and home-level changes.

A data and safety monitoring board with expertise in geriatric medicine and clinical trials met twice to review trial processes. Informed consent was obtained from a representative at each LTC home and from individual professionals. The study was approved by the McMaster University/Hamilton Health Sciences Research Ethics Board (Reference #:09–215).

### Recruitment of clusters

Our sampling frame was LTC homes who received medication and consulting services from Medical Pharmacies Group Limited, a large provider delivering services to approximately one-third of Ontario LTC homes. Homes were eligible if they had more than one prescribing physician and received services from Medical Pharmacies. There were no resident-level exclusion criteria. Our recruitment strategy included homes located in communities of all sizes and geographical regions across Ontario. Recruitment began in 2009 and was ongoing until the target sample size was reached. The final home completed the intervention in 2012.

### Participants

The target audience within each LTC home was the core group of interdisciplinary care leaders (that is, the professional advisory committee). Depending on the size of the LTC home, this group included the medical director, director/assistant director of care, administrator, consultant pharmacist, food services director, other prescribing physicians, nurse practitioners, registered nurses, dieticians, and physiotherapists. Participation in the ViDOS intervention sessions is fully described elsewhere [18].

### Randomization and blinding

LTC homes were allocated to control or intervention arms (1:1 allocation ratio) using stratified, block randomization. Stratified allocation was based on home size (<250 versus ≥250 beds) and profit/nonprofit status. An offsite investigator assigned homes to treatment groups based on a computer-generated allocation sequence. The database manager and analysts were blinded to allocation status; LTC homes, experts, and coordinators were not blinded.

### Intervention

**Figure 2:**
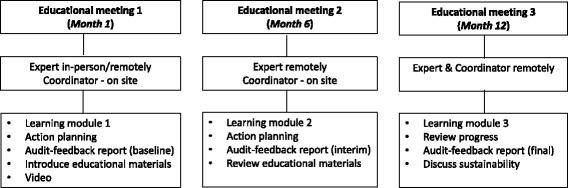
ViDOS multifaceted intervention.

The design and implementation of our 12-month, multifaceted intervention (Figure 2) was founded on the Canadian Institutes of Health Research Knowledge-to-Action cycle [21], described in the study protocol [19].

#### Educational meetings

Informal “champions” (typically Directors of Care) worked with the research team to book educational meetings and encourage participation. Educational meetings had a combination of didactic presentation and interactive activities. Three, small-group, educational meetings, were held at each intervention home during approximately months 1, 6 and 12. Meetings were one-hour in length, typically had 5 to 10 participants, and were facilitated by one of six expert opinion leaders^a^ [22], who were specialist physicians with expertise in osteoporosis or geriatrics. Experts engaged with study participants either in-person (meeting one only) or remotely; the study coordinator was on-site at the first two meetings to distribute/collect study materials and to facilitate the process (for example, connecting with the expert if remotely conducted). At each meeting, the expert delivered a standardized presentation reviewing best practices for osteoporosis management and fracture prevention (including case studies) with an emphasis on the importance of vitamin D for preventing falls and fractures. A question and answer session followed the presentation.

#### Action planning for quality improvement

After the presentation, interdisciplinary teams engaged in action planning based on the “plan-do-study-act” cycle [19,23]. Interdisciplinary care teams brainstormed regarding barriers and facilitators to implementing evidence-based strategies, particularly changes that could be made at an organizational level. An action plan worksheet was completed/updated at each educational meeting, which outlined specific tasks and steps for implementing process/policy changes. Ideas generated by teams were shared among LTC homes.

#### Audit and feedback reports

During the session, experts reviewed home-level audit and feedback reports with the interdisciplinary team. Reports included 1) vitamin D, calcium, and osteoporosis medication prescribing (based on previous month), which was benchmarked against other study homes; and 2) a summary of the falls and fractures occurring in the home in the previous 3 months. Confidential audit and feedback reports were also provided to each physician, containing a prescribing summary for their roster of patients.

#### Educational materials

Educational materials distributed included *Ontario Osteoporosis Strategy* [24] fracture prevention toolkits (*ibid*), process checklists, and paper-based treatment alerts to assist consultant pharmacists with flagging high-risk individuals [19].

#### Changes to intervention

The expert attended the initial educational meeting in-person at the first seven homes; it was not feasible to continue with this format, and the remainders were conducted remotely. We planned to use webinar technology to conduct the educational meetings; however, many homes did not have an accessible internet connection, and experts facilitated meetings via teleconference instead.

#### Control arm

Control homes received no intervention except fracture prevention toolkits provided to all LTC homes in the province by the *Ontario Osteoporosis Strategy* [24]; Osteoporosis Long-term care [www.osteoporosislongtermcare.ca].

### Outcomes

Resident-level, de-identified prescribing/clinical data were obtained from the Medical Pharmacies central database. Data captured were point prevalence estimates; that is, medication/supplementation orders for all residents residing in the home on the day of the data download. Data were downloaded separately for each LTC home at approximately baseline, 6 and 12 months, just prior to the scheduled ViDOS educational session^b^. To ensure that the timing of data downloads was balanced between study arms throughout the study, data downloads for control homes were chronologically matched with an intervention home (the nearest one in the randomization sequence).

The primary outcome was the change in the proportion of all residents prescribed vitamin D ≥800 IU/day (including vitamin D2 or D3) over 12 months. Secondary prescribing outcomes were the change in 1) the proportion of all residents prescribed calcium ≥500 mg/day and 2) high-risk residents prescribed an osteoporosis medication (oral bisphosphonate, zoledronic acid, denosumab, or teriparatide). Algorithms to calculate supplement dosage included all daily/weekly/monthly preparations and medications and vitamin/mineral supplements that contain vitamin D and calcium. High-risk residents were those with a documented hip fracture, vertebral fracture, or osteoporosis diagnosis on the electronic Medication Administration Record (eMAR). The eMAR captured any medication indications or diagnoses that were present at admission, and further updates may have occurred when diagnoses were included on physician orders or quarterly medication reviews.

#### Falls and fractures

The study was not powered to make comparisons between study arms regarding incident falls and fractures. These data were collected to inform the feasibility of data collection for future trials in this setting. Falls and fracture data were collected for 3 months, in three nonconsecutive periods, coinciding with the educational meetings for the intervention homes. Home-specific feedback on the number of falls and fractures occurring was included in the audit and feedback reports (intervention homes only). Researchers provided the homes with a standardized data collection sheet and homes completed the information using various sources including electronic/paper-based charts, internal monitoring systems, Resident Assessment Instrument - Minimum Data Set 2.0 (RAI-MDS 2.0), and critical incident reports.

### Sample size calculation

Given effect sizes observed in other KT interventions (for example, mixed interactive and didactic educational meetings [25]), we were interested in detecting a 15% difference in prescribing of vitamin D ≥800 IU/daily between the groups (anticipating a 20% increase in the intervention group, and a 5% increase in the control group due to ongoing provincial initiatives [24]). Based on prior work, we anticipated an average of 120 residents per LTC home and a baseline vitamin D (≥800 IU/day) prescribing rate of 30% [11]. Assuming an intracluster correlation of 0.10 and a Type I error of 5%, we determined that a sample size of 18 LTC homes (n = 2,160 residents) per arm was required to achieve 82% power. Factoring in a 10% dropout rate, the recruitment target was 40 LTC homes.

### Statistical analysis

Differences in baseline characteristics between arms were examined using the chi-square procedure and independent samples *T*-test. Our primary analysis was intention-to-treat (ITT). To account for clustering within a LTC home, we analyzed resident-level data using the generalized estimating equations (GEE) technique, assuming an exchangeable correlation structure which specifies that all observations within the same cluster are equally correlated [26]. We examined the effect of the intervention on the change from baseline to 12 months in the proportion of residents prescribed vitamin D ≥800 IU/day and calcium ≥500 mg/day (that is, treatment group-by-time interaction). The same method was used to examine osteoporosis medication prescribing, including only high-risk residents. Odds ratios (ORs), corresponding 95% confidence intervals (CIs) are reported.

#### Sensitivity analyses

The above analyses were also conducted in the per protocol cohort, which excluded non-active intervention homes. We also examined the effect of adjusting GEE models for age, sex, and high-risk status.

#### Cluster-level analyses

For each outcome, we calculated the absolute prescribing change in each LTC home from baseline to 12-months. We report mean home-level prescribing changes within treatment arms and calculated the difference between arms, with 95% CIs adjusted for clustering using the method described by Donner and Klar [27].

All statistical analyses were performed using the SAS/STAT 9.2 software package (SAS Institute Inc., Cary, NC, USA) and SPSS v20. The criterion for statistical significance was set at ɑ = 0.05.

## Results

The CONSORT flow diagram is displayed in Figure 1. The baseline cohort consisted of 5,478 residents residing in 40 LTC homes (19 intervention, 21 control). In total, 12 of the 19 intervention homes actively participated in the intervention. Of the seven nonactive intervention homes, six withdrew participation before beginning the study and one withdrew after the first educational meeting. The primary reasons for not participating were logistical or scheduling difficulties [18].

### Facility characteristics

The majority of LTC homes in the study were for-profit and affiliated with a multifacility chain (Table 1). The mean facility size was larger in control (157 beds, standard deviation (SD) 80.2) versus intervention homes (115 beds, SD 67.9); however, both study arms had a similar proportion of small (<100 beds) and large (>250 beds) homes.

**Table 1:** **Baseline characteristics of intervention and control long-term care homes**

**Characteristic**	**Control**	**Intervention**
	**(n = 21)**	**(n = 19)**
Facility size (number of beds) mean (SD), min, max	157 (80.2), 49, 375	115 (68.0), 43, 294
Number of prescribing physicians mean (SD), min, max	4.3 (2.7), 2, 13	4.5 (2.7), 1, 10
Percent (n) of LTC homes located in communities of various population sizes		
Small (<30,000)	33 (7)	47 (9)
Medium (30,000 - 100,000)	10 (2)	11 (2)
Large (100,000-1,000,000)	38 (8)	26 (5)
Metropolitan (>1,000,000)	19 (4)	16 (3)
For-profit, %	81	95
Chain affiliation, %	76	84

SD, standard deviation; min, minimum value; max, maximum value.

### Resident characteristics

Residents in both arms were similar in baseline demographic characteristics. In the control arm, there was a higher prevalence of hip fractures; osteoporosis diagnoses; and baseline use of vitamin D ≥800 IU/day, calcium ≥500 mg/day, and osteoporosis medications (Table 2). Due to the correlated nature of clustered data and typically smaller number of units being randomized, achieving balance in baseline characteristics is less likely in cluster trials compared with individual RCT’s [28].

**Table 2:** **Baseline characteristics of residents in intervention and control long-term care homes**

**Characteristic**	**Control group**	**Intervention group**
	**n = 3,293**	**n = 2,185**
Age, mean (SD)	84.6 (10.7) (n = 3,274)	84.0 (11.1) (n = 2,178)
Female, % (n)	71.1 (2,329/3,277)	70.4 (1,532/2,175)
Number of medications, mean (SD)	9.2 (4.3) (n = 3,293)	9.7 (4.7) (n = 2,185)
Vitamin D (≥800 IU/day), % (n)	41.8 (1,378/3,293)	36.0 (787/2,185)
Calcium (≥500 mg/day), % (n)	34.8 (1,145/3,293)	30.7 (671/2,185)
Osteoporosis medication, % (n)	22.7 (747/3,293)	17.0 (372/2,185)
High-risk residents,* % (n)	34.8 (412/1,185)	26.7 (181/678)
Hip fracture (prevalent), % (n)	7.0 (230/3,290)	5.0 (109/2,183)
Vertebral fracture (prevalent), % (n)	1.4 (46/3,290)	0.9 (20/2,183)
Osteoporosis diagnosis, % (n)	31.3 (1030/3,290)	27.8 (607/2,183)
High-risk, % (n)*	36.0 (1185/3,293)	31.0 (678/2,185)

SD, standard deviation; *Hip fracture, spine fracture, or osteoporosis diagnosis.

### Prescribing changes

The median lengths of follow-up between baseline and final data download for all intervention, active intervention, and control homes, respectively, were 12.4 (min 7.4, max 15.0)^c^, 12.2 (min 11.4, max 15.0), and 12.1 (min 10.5, max 13.4) months.

The main findings are presented in Table 3. In the ITT cohort, GEE analyses indicated there was significantly greater prescribing change from baseline to 12 months in the intervention versus control groups for both vitamin D and calcium, with ORs 1.82 (95% CI: 1.12, 2.96) and 1.33 (95% CI: 1.01, 1,74), respectively.

**Table 3:** **Effect of ViD** ***OS*** **intervention on prescribing outcomes**

**Outcome**	**OR (95% CI)**	
	**Unadjusted**	Adjusted*
**ITT cohort (n = 21 control, n = 19 intervention)**		
Primary: Vitamin D (≥800 IU/day)	1.82 (1.12, 2.96)	1.85 (1.13, 3.06)
Secondary: Calcium (≥500 mg/day)	1.33 (1.01, 1.74)	1.33 (1.01, 1.77)
Osteoporosis medication (high-risk residents**)	1.17 (0.91, 1.51)	1.12 (0.87, 1.44)
**Per protocol cohort (n = 21 control, n = 12 intervention†)**		
Primary: Vitamin D (≥800 IU/day)	3.06 (2.18, 4.29)	3.14 (2.22, 4.45)
Secondary: Calcium (≥500 mg/day)	1.57 (1.12, 2.21)	1.58 (1.11, 2.24)
Osteoporosis medication (high-risk residents**)	1.20 (0.90, 1.60)	1.16 (0.87, 1.53)

*Adjusted for age, sex and high-risk status (hip fracture, spine fracture, or osteoporosis diagnosis). **Hip fracture, spine fracture, or osteoporosis diagnosis.

† Long-term care homes that were active participants in the intervention.

ITT, intention to treat; OR, odds ratio; CI, confidence interval.

The intervention had no significant effect on the change in osteoporosis medication prescribing in high-risk residents (OR 1.17, 95% CI: 0.91, 1.51).

The intracluster correlation coefficients for vitamin D, calcium, and osteoporosis medication prescribing were 0.194, 0.112, and 0.052, respectively.

#### Sensitivity analyses

In the per protocol cohort (including only actively participating homes), ORs were 3.06 (95% CI: 2.18, 4.29), 1.57 (95% CI: 1.12, 2.21), and 1.20 (95% CI: 0.90, 1.60) for vitamin D, calcium and osteoporosis medications, respectively.

Adjustment for confounding (age, sex, and high-risk status) had little impact on effect estimates (Table 3).

#### Absolute prescribing change

Over the course of the trial, the mean home-level prescribing change for vitamin D ≥800 IU/day was 22.2% (95% CI: 17.6, 26.7) in the intervention arm versus 7.5% (95% CI: 5.7, 9.3) in the control arm (between group difference = 14.7%, 95% CI: 13.1, 16.2). Mean home-level prescribing change for calcium ≥500 mg/day was 8.8% (95% CI: 6.6, 11.0) in the intervention arm versus 1.8% (95% CI: 0.30, 3.24) in the control arm (between group difference = 7.0%, 95% CI: 6.2, 7.9). In the per protocol cohort, the difference in mean home-level prescribing change between treatment arms was 27.0% (95% CI: 25.5, 28.5) for vitamin D and 13.1% (95% CI: 12.0, 14.2) for calcium.

There was no significant difference in the home-level prescribing change between arms for osteoporosis medications (between group difference, ITT = 3.4%, 95% CI: 2.6, 4.2; per protocol = 2.9%, 95% CI: 1.7, 4.1).

### Falls and fractures

Complete falls and fracture data were received from 18 control (baseline residents, n = 2,727) and 11 intervention (baseline residents, n = 1290) homes. During 9 months of data collection (three nonconsecutive periods), 18 control homes (baseline residents, n = 2,727) reported 1,712 fallers (43.6% single fall, 19.3% two falls, and 37.1% =3 falls) and 11 intervention homes (baseline residents, n = 1290) reported 853 fallers (44.4% single fall, 19.0% with two falls, and 36.6% with three falls). In the control and intervention groups, respectively, 79/5,128 (1.5%) and 41/2,491 (1.6%) of all reported falls resulted in a fracture, including 40/79 (50.6%) and 17/41 (41.5%) hip fractures.

## Discussion

Our results suggest that the ViDOS interdisciplinary, multifaceted intervention resulted in significantly greater uptake of appropriate vitamin D and calcium prescribing, with an absolute improvement in prescribing over 12 months of approximately 15% for vitamin D and 7% for calcium in the ITT cohort. Although not the focus of this report, we were also interested in the feasibility of conducting this type of KT intervention in the LTC setting. Despite challenges associated with recruitment and retention, there was good participation by interdisciplinary team members, including physicians, pharmacists and Directors of Care, and the intervention was well received [18].

Given that it is a tolerable, low-cost intervention that is recommended for all older adults [29], vitamin D may be particularly amenable to targeted change. In the community, a multifaceted intervention targeting improved osteoporosis management demonstrated a 13% absolute improvement in vitamin D [30]. In LTC, one KT intervention involving consultation and training by specialist osteoporosis nurses demonstrated a relative increase of 64% in calcium and vitamin D prescribing [31], but another study with a similar multifaceted intervention to ours did not demonstrate significant effects [32]. In the latter study, participation in the intervention was low, only high-risk residents were included in the analysis, and the authors also suggest a possible ceiling effect due to high baseline prescribing rates.

We did not see a significant effect for osteoporosis medication prescribing, which we hypothesize is attributable to several factors. Management with osteoporosis medications was reviewed in the didactic component of the educational meetings (the standardized presentation by the expert opinion leader), and this format has been demonstrated as less effective [33]. In the interactive component, action planning for change, several process and policy changes were discussed that may have been more amenable to vitamin D and calcium (for example, standard orders, dietary review and changes). It has been well documented that family physicians face a number of barriers for prescribing osteoporosis medications in LTC [10,14], including difficulty in applying general osteoporosis guidelines to LTC residents, particularly with regard to risk assessment. Currently, general osteoporosis clinical practice guidelines [29], are being adapted for the frail elderly residing in LTC. Furthermore, providing information and recommendations at the point of care that incorporate fracture risk may be necessary components for enhancing uptake in osteoporosis medications and is an area for future research.

One of the strengths of our multifaceted KT intervention was the inclusion of content and strategies that encouraged organizational change specific to the LTC setting, in addition to targeting professional behavior change. This is an important consideration, given that several structural and environmental barriers for implementing evidence-based practices within LTC homes have been identified including a high proportion of unregulated staff, absence of a learning culture, high turnover in management, heavy regulatory and documentation demands, routinized care rituals, and lack of familiarity with clinical practice guidelines [34-36]. Although not all barriers are easily modifiable, the ongoing monitoring of organizational-level barriers (formally, three times over 12 months) was an important design feature.

Another important design component was tailoring our intervention to interdisciplinary care teams. Compared with other practice settings, physicians who practice in LTC are typically more removed from daily patient care and more reliant on collaborative decision making. We engaged the entire interdisciplinary care team, including physicians, by scheduling educational meetings in conjunction with quarterly professional advisory meetings, enabling several off-site professionals to be present simultaneously.

Although not the focus of this paper, we had good fidelity with our intervention including active participation in educational meetings. Other similar KT studies in LTC, which included both physicians and LTC staff may have had nonsignificant [15,37] or less than optimal results [38], due to poor adherence with educational components. In addition to scheduling educational meetings with regularly scheduled meetings, feedback from ViDOS participants indicated that the direct involvement of an expert was highly valued [39].

This pilot study provides evidence that KT interventions can be successfully applied within the LTC setting, particularly when structured to fit the unique practice environment and organizational structure. Previously, these KT strategies have mainly been evaluated in non-LTC practice settings, and studies demonstrate small to moderate effectiveness [40]. Cochrane systematic reviews indicate median absolute improvements in care in the range of 4 to 12% for educational meetings (including interactive and didactic [25]), educational outreach [41], local opinion leaders [42], audit and feedback [43], and computerized reminders [44]. Some organizational interventions (for example, multidisciplinary collaboration and knowledge management change) also appear to improve some care outcomes [45], and interprofessional education has shown some positive results, but is an area requiring further study [46]. We observed similar or larger effect sizes compared with the medians reported in the systematic reviews noted above.

The ViDOS study had several methodological strengths, including the recruitment of homes that were geographically diverse and located in communities of varied population sizes. Both the study design and analysis took into the account clustered nature of the data, which minimizes the possibility of overestimating the treatment effect and spuriously significant findings [47].

Nonetheless, our study is not without limitations. While our study was generalizable in terms of geography and community size, we had an over-representation of chain affiliated and for-profit LTC homes compared with provincial averages [48]. Not-for-profit LTC homes have been associated with higher quality of care [49], although multifacility chains may have greater resources to facilitate implementation of clinical practice guidelines [34]. We experienced some challenges with recruitment and retention, which has also been noted in other KT trials in LTC [31,50]. This was a pragmatic RCT; some contamination between study arms likely occurred, which could have diluted our treatment effect. Six consultant pharmacists and four Medical Directors practiced in both control and intervention homes, and control homes may have been impacted by study activities via diffusion of messages (for example, homes in the same corporate chain). Future trials should consider adding region as a stratification factor to minimize contamination. Both arms were subject to outside influences including an ongoing province-wide initiative, the *Ontario Osteoporosis Strategy for LTC*, [www.osteoporosislongtermcare.ca) [24]].

We collected incident falls and fracture data for feasibility data, and the ability to obtain standardized data from LTC homes was a noted methodological limitation due to different fall reporting systems. Our fracture rate was slightly lower than the rate observed in a Canadian study using a standardized fall risk surveillance tool [51]. However, the purpose of this pilot study was to examine the feasibility and effectiveness of a KT model, and not to demonstrate the effectiveness of vitamin D for reducing falls and fractures. The choice of Vitamin D as our primary outcome was based on previous studies that demonstrated falls and fracture reduction [8,9] and our scoping review [52], which indicated that in LTC vitamin D supplementation was the intervention with the strongest evidence for reduction of hip fractures.

Given that our intervention was multifaceted, it is difficult to determine the most influential components. In general, multifaceted interventions may be more effective than single interventions [53], although Grimshaw *et al*. [40], have shown that adding more strategies may not improve effect sizes. We did not include measures of organizational context (for example, work culture, and type of leadership), which have been identified as an important factor influencing the uptake of research evidence [54]. Future trials should consider the interplay with contextual factors.

## Conclusions

Although RCT’s evaluating KT interventions are increasing in the LTC setting [31,32,37,55], further implementation research evaluating professional and organizational KT strategies is still greatly needed. In this study, we demonstrated that an interdisciplinary, multifaceted intervention could be feasibly implemented in LTC [18] and that it improved uptake of vitamin D and calcium prescribing. Although our topic focused specifically on osteoporosis and fracture prevention, the ViDOS model could potentially be applied to a wider range of topics relevant to LTC residents.

## Endnotes

^a^In the framework by Locock *et al*. [18], an expert opinion leader was considered distinct from peer opinion leaders who are role models in daily practice and was a ‘credible authority (often an academic or consultant) able to explain the evidence and respond convincingly to challenges and debate’.

^b^Our rationale for linking data downloads with ViDOS session dates was to generate up-to-date audit and feedback reports. Six-month data were used to generate interim audit and feedback reports only, not for outcome evaluation.

^c^In homes that were nonactive participants, there were additional challenges in obtaining the data. For two nonactive intervention homes, the elapsed time between baseline and a follow-up data download was less than 8 months. In the remaining homes, the time between baseline and follow-up ranged from 11 to 15 months. The variability in prescribing download dates was due to the timing of the data downloads with educational sessions.

## Abbreviations

ViDOS: vitamin D and osteoporosis study
LTC: long-term care
KT: knowledge translation
RCT: randomized controlled trial
OR: odds ratio
SD: standard deviation
eMAR: electronic Medication Administration Record
CI: confidence interval
GEE: generalized estimating equations
ITT: intention-to-treat
